# Alterations in Postural Control during the World's Most Challenging Mountain Ultra-Marathon

**DOI:** 10.1371/journal.pone.0084554

**Published:** 2014-01-21

**Authors:** Francis Degache, Jérôme Van Zaen, Lukas Oehen, Kenny Guex, Pietro Trabucchi, Gégoire Millet

**Affiliations:** 1 School of Health Sciences, Health Research Unit, University of Applied Sciences Western Switzerland, Lausanne, Switzerland; 2 Applied Signal Processing Group, École Polytechnique Fédérale de Lausanne, Lausanne, Switzerland; 3 Institute of Sports Sciences, Department of Physiology, Faculty of Biology and Medicine, University of Lausanne, Lausanne, Switzerland; 4 School of Health Sciences, Department of Physiotherapy, University of Applied Sciences Western Switzerland, Lausanne, Switzerland; 5 Faculty of Sports Sciences, University of Verona, Verona, Italy; The University of Queensland, Australia

## Abstract

We investigated postural control (PC) effects of a mountain ultra-marathon (MUM): a 330-km trail run with 24000 m of positive and negative change in elevation. PC was assessed prior to (PRE), during (MID) and after (POST) the MUM in experienced ultra-marathon runners (n = 18; finish time = 126±16 h) and in a control group (n = 8) with a similar level of sleep deprivation. Subjects were instructed to stand upright on a posturographic platform over a period of 51.2 seconds using a double-leg stance under two test conditions: eyes open (EO) and eyes closed (EC). Traditional measures of postural stability (center of pressure trajectory analysis) and stabilogram-diffusion analysis (SDA) parameters were analysed. For the SDA, a significantly greater short-term effective diffusion was found at POST compared with PRE in the medio-lateral (ML; *Dxs*) and antero-posterior (AP) directions (*Dys*) in runners (p<0.05) The critical time interval (*Ct*x) in the ML direction was significantly higher at MID (p<0.001) and POST (p<0.05) than at PRE in runners. At MID (p<0.001) and POST (p<0.05), there was a significant difference between the two groups. The critical displacement (*Cdx*) in the ML was significantly higher at MID and at POST (p<0.001) compared with PRE for runners. A significant difference in *Cdx* was observed between groups in EO at MID (p<0.05) and POST (p<0.005) in the ML direction and in EC at POST in the ML and AP directions (p<0.05).

Our findings revealed significant effects of fatigue on PC in runners, including, a significant increase in *Ctx* (critical time in ML plan) in EO and EC conditions. Thus, runners take longer to stabilise their body at POST than at MID. It is likely that the mountainous characteristics of MUM (unstable ground, primarily uphill/downhill running, and altitude) increase this fatigue, leading to difficulty in maintaining balance.

## Introduction

Postural control is a complex function that involves maintaining the projection of the centre of mass (*CoM*) within the base of support [1]. The maintenance of equilibrium is achieved using orientation information derived from 3 independent sensory sources: somatosensory, vestibular and visual inputs. The **3** sensory systems all contribute to postural control, and damage to any or to the brainstem or cerebellum will influence the overall output of the postural system [1]. Postural control is also dependent upon reflexive and voluntary muscle responses [2]. Balance is actively controlled by the central nervous system, which calls into action various relevant postural muscles when they are required [3]. In a healthy individual, postural strategy appears to choose an equilibrium state with small-amplitude oscillations while keeping the body close to vertical alignment [4]. The bipedal control of posture in the Eyes Opened (EO) or Eyes Closed (EC) conditions has primarily been studied by analysing the trajectory of the centre of pressure (*CoP*), which can be easily recorded using posturographic platforms [5].

A deterioration in postural control due to muscular fatigue has been reported in several studies [6]–[17]. Fatigue induced by exercise is determined by a combination of physiological processes that occur at both central and peripheral levels and that result in an inability to produce an expected force level and an increase in the onset delay of postural control movements [12], [18], [19]. The effect of prolonged exercise on postural control has been investigated in both trained [20] and untrained subjects [21]. All studies showed a decrease in the ability to balance after fatiguing exercise.

It has also been shown that postural stability is affected by >19, 24 or 48 h of sleep deprivation (SDep) [22]–[25], although the underlying mechanisms have not yet been determined. Studies on the modifications of postural control during periods of sustained wakefulness have reported that it varied based on attention levels [22]–[27]. In fact, deterioration in cognitive function caused by fatigue could be predicted by a short posturographic test [22]. This latter test has even been proposed as a mean to assess fatigue levels due to SDep [26]. After a complex and dynamic analysis of data, results correlated significantly with physiological markers and validated cognitive measures of fatigue. In this study, we did not use the third condition on a soft support. After MUM, kinaesthetic afferences are greatly altered due to foot/toe injuries (e.g., blisters and oedemas). Due to time constraints (e.g., mid-measurements performed during the race), we choose not to test these influences.

Ultra-endurance running or the ultra-marathon (e.g., over a distance longer than the traditional marathon) represents a unique opportunity and is an excellent model for investigating the effects of extreme exercise [28]. Mountain ultra-marathons (MUMs; e.g., Western States 100, Ultra-trail du Mont-Blanc, Tor des Géants) induce specific challenges (e.g., large changes in elevation that induce muscle fatigue/damage, SDep, altitude) because they comprise running/walking on mountain trails with positive and negative slopes. Therefore, MUMs have been associated with sleep deprivation [29], [30], critical muscle damage and general fatigue [30], [31]. Some previous studies have already assessed the acute consequences of MUM on inflammation, neuromuscular and cardiac fatigue or haemolysis [30]–[35], but none have assessed the consequences of such an extreme event on postural control. Interestingly, each of the characteristics (e.g., muscle fatigue/damage, SDep, altitude) of MUM is likely to impair postural stability to a large extent. In this study, we aimed to investigate the aetiology of postural control and somatosensory integration measured using posturographic tests in the most extreme MUM in the world. By using data recorded prior to, during and after the event on both runners and non-exercising control subjects submitted to the same level of SDep, we aimed to better dissociate the alterations induced by muscle fatigue/damage from the alterations induced by SDep fatigue. We tested the hypothesis that postural alterations would be influenced to a greater degree by muscle fatigue, specific to the mountainous characteristics of MUM, than by sleep deprivation.

## Materials and Methods

This experiment is part of a large research project incorporating running mechanics and neuromuscular fatigue assessment. Thus, some parts of the methods and results have been reported elsewhere [30], but they are repeated here for the convenience of the reader.

### Subjects

Eighteen male runners participated in this study. They were aged 44.0±10.7 years, measured 173.7±4.8 cm tall and weighed 68.7±5.6 kg at the start of the race. From the twenty-five initially engaged participants, 18 subjects participated in the three testing sessions. All subjects were experienced in ultra-marathon/trail running, trained 7.2±4.1 h per week and had 7.8±6.9 years of experience in trail running. Nine had already finished the previous Tor des Géants (TdG). In addition, a control group of 8 non-runner subjects participated in this study. They were aged 29.3±8.1 years (significantly different from the runners group, p<0.05), measured 174.1±5.6 cm tall and weighed 70.9±9.3 kg. The control group underwent the same sleep deprivation and the same tests as the runners. The instructions for sleep deprivation to the control group were as follows: “Sleep as little as possible, and if you sleep, record your sleeping duration”.

All changes in weight and body fat during the race/study are presented in Table 1.

**Table 1:** Main characteristics of the runners and the control group.

Group		Age	Mass	Body fat	Sleep
					duration
		(yrs.)	(kg)	(%)	(h)
**Runners**	PRE		68.7±5.6	17.8±3.3	-
**n = 18**	MID	44.0±10.7	68.5±5.0	17.5±3.3	1.3±1.7
	POST		68.2±0.0	18.4±3.2	8.6±5.3
**Control**	PRE		70.9±9.3	20.1±6.1	-
**n = 8**	MID	29.3±8.1***	70.7±8.9	19.2±6.5	1.2±1.8
	POST		71.0±8.4	19.7±6.2	12.3±5.4

***p<0.001 compared with Runners.

### Ethics Statement

All subjects were fully informed of the procedure and the risks involved. They all provided written consent. They were allowed to stop the study at will and to refuse any of our tests.

This study was approved by the institutional ethics committee of the University of Verona, Italy (Approval #152, Department of Neurological, Neuropsychological, Morphological and Motor Sciences). All subjects provided written, voluntary, informed consent prior to participation. The experiment was conducted according to the Declaration of Helsinki.

### Experimental design

**Figure 1:**
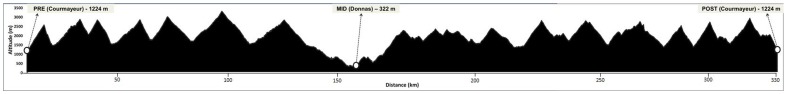
GPS track of the entire run with the three test session locations and the distance scale in km.

The race supporting this study was the Tor des Géants. It comprises running/walking 330 km with a total positive and negative elevation of 24000 m (Fig. 1). This race is considered to be the world's most challenging single-stage MUM. The maximum and minimum altitudes are 3300 m and 322 m, respectively. There are 20 passes over 2000 m. The distance is divided into seven parts, with six interspersed aid stations where sleeping is allowed. However, the participants do not have to make any compulsory stop and therefore can pace themselves and manage their stops as they wish. Because the recovery time (nutrition, hydration, sleep, etc.) is not subtracted from the race time, the influence of pacing and SDep is of paramount importance. This race is therefore different from other MUMs of shorter distances (e.g., Ultra Tour du Mont-Blanc (UTMB), 166 km [31]) and from road ultra-marathons over longer distances but with several stages (e.g., Trans Europe Foot Race, 4,487 km in 64 stages from South Italy to North Cape, Norway in 2009, [36]), where sleep management is of less importance.

The runners and control subjects were tested three times: prior to the run (PRE, Courmayeur, Italy, altitude 1224 m, km 0), during the run (MID, Donnas, Italy, altitude 322 m, km 148.7) and during the 30 min following completion of the run (POST, Courmayeur, Italy, altitude 1224 m, km 330).

### Experiments

#### Postural stability protocol

During three experimental sessions, a posturographic platform (Fusyo-Medicapteur, Toulouse, France; Dekra certification) at a 40-Hz sampling rate was used. *CoP* data were recorded using a PC computer with Fusyo software (V1.2.1 - Medicapteur, Toulouse, France). The posturographic platform measured 530 mm×460 mm×35 mm and was equipped with three pressure gauges (hysteresis <0.2%). Signal processing was accomplished using a 16-bit A/D converter at 40 Hz [37]. The duration of each test was 51.2 seconds, resulting in a 2048-point time series.

The subjects were placed based on precise markers. Their legs were extended, and their feet formed a 30° angle relative to each other with an inter-malleolar distance of 5 cm. The subjects were first instructed to maintain their balance in the EO and then EC condition. In the EO condition, the subjects looked at a fixed-level target at a distance of 90 cm. They were instructed to stand with their arms at their sides and to look straight ahead. The height of the visual target was adjusted for each subject. They were instructed to stand double-leg on the platform while trying to maintain postural stability during the trials in all sessions. While in the EC condition, they were asked to keep their gaze straight ahead and to maintain postural stability. The verbal instructions were as follows: “Stand with your arms at your sides and look straight ahead while trying to maintain your postural stability to your best ability”. The intention of the instructions was to ask the participants not to fall and to limit their sway as much as possible. Only one postural test was performed in each condition (EO and EC). To limit potential recovery time, no familiarisation session was conducted.

#### Perceived exertion

The fatigue sensations were measured at the PRE, MID and POST sessions using a 10-cm visual analogue scale (VAS) [38]. The subjects were instructed to report their general feelings of fatigue.

### Data acquisition and analyses

*CoP* data were collected to extract the standard postural sway parameters: (1) total displacement of *CoP* in AP and ML (length in mm); (2) mediolateral displacement of *CoP* (mm; ML-sway range); and (3) anteroposterior displacement of *CoP* (mm; AP-sway range). These parameters were computed for each 51.2-second trial and then averaged for each set of 10 trials to obtain an average value for each parameter and for each subject in each experimental condition. Dependence on vision was analysed using the Romberg coefficient that is the total *CoP* displacement measurement in the EC condition divided by the total *CoP* displacement measurement in the EO condition [39]. In addition, stabilogram-diffusion analysis (SDA) [5], [40] was performed for each subject, each recording time and each condition. First, the squares of the displacements between all pairs of *CoP* points (ML and AP) separated by a specified time interval were computed [5]. This analysis was repeated for time intervals ranging from 1/40 to 10 second in steps of 1/40 second. The squared displacements were then averaged for each time interval. Finally, the mean squared displacements were plotted versus the time intervals, resulting in stabilogram-diffusion plots. These plots were obtained for ML, AP and planar displacements. The SDA parameters were extracted by fitting two linear regressions on the stabilogram-diffusion plots: one in the short-term region (for time intervals smaller than or equal to 0.5 second) and one in the long-term region (for time intervals between 2 and 10 second). The regressions yielded the following parameters: the diffusion coefficients for short- and long-term regions (*Ds* and *Dl*) and the coordinates of the intersection of the two regression lines (*Ct* and *Cd*), also known as the critical point. The diffusion coefficients are defined as half the slope of the corresponding regressions. A third-order Butterworth low-pass filter was applied to the positional data on markers using a built-in MATLAB function.

**Figure 2:**
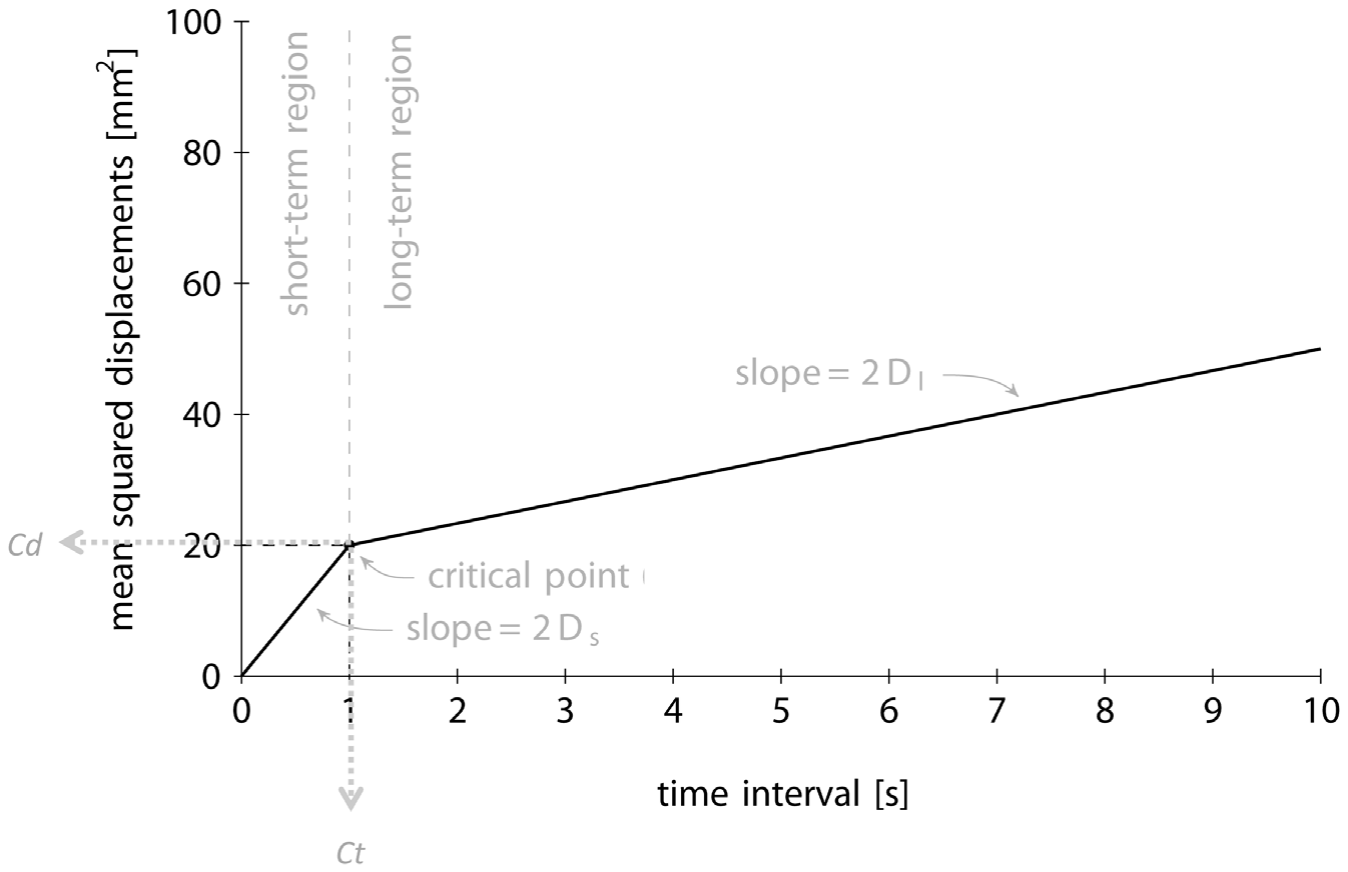
Description of Stabilogram-diffusion slope.

All SDA parameters are depicted in Figure 2.

### Statistical analyses

All data presented in the text, tables and figures are the mean value ± SD. Data were screened for normality of distribution and homogeneity of variance using Shapiro-Wilk normality tests and Barlett's tests, respectively.

To compare means for each dependent variable (the standard postural sway parameters and the SDA parameters) between the three independent experimental sessions (PRE, MID, POST) and between both groups (runners vs. controls), we used a two-way repeated measures ANOVA for each dependent variable followed by post-hoc Tukey tests.

For all statistical analyses, a P value of 0.05 was accepted as the level of significance. Statistical analyses were computed using commercially available software (SigmaPlot version 9.0; Systat Software, CA).

## Results

### Performance, sleep deprivation and perceived level of fatigue

The average finishing time of the subjects was 126 h 40 min ±16 h 49 min (final rank from 7^th^ to 243^th^ position out of 301 finishers). The time between arrival (finish line) and the POST measurements was 18±06 min.

Sleep duration was not different between the controls and the runners at MID (1.3±1.7 vs. 1.2±1.8 h) and at POST (8.6±5.3 vs. 12.3±5.4 h). The results are presented in Table 1.

**Figure 3:**
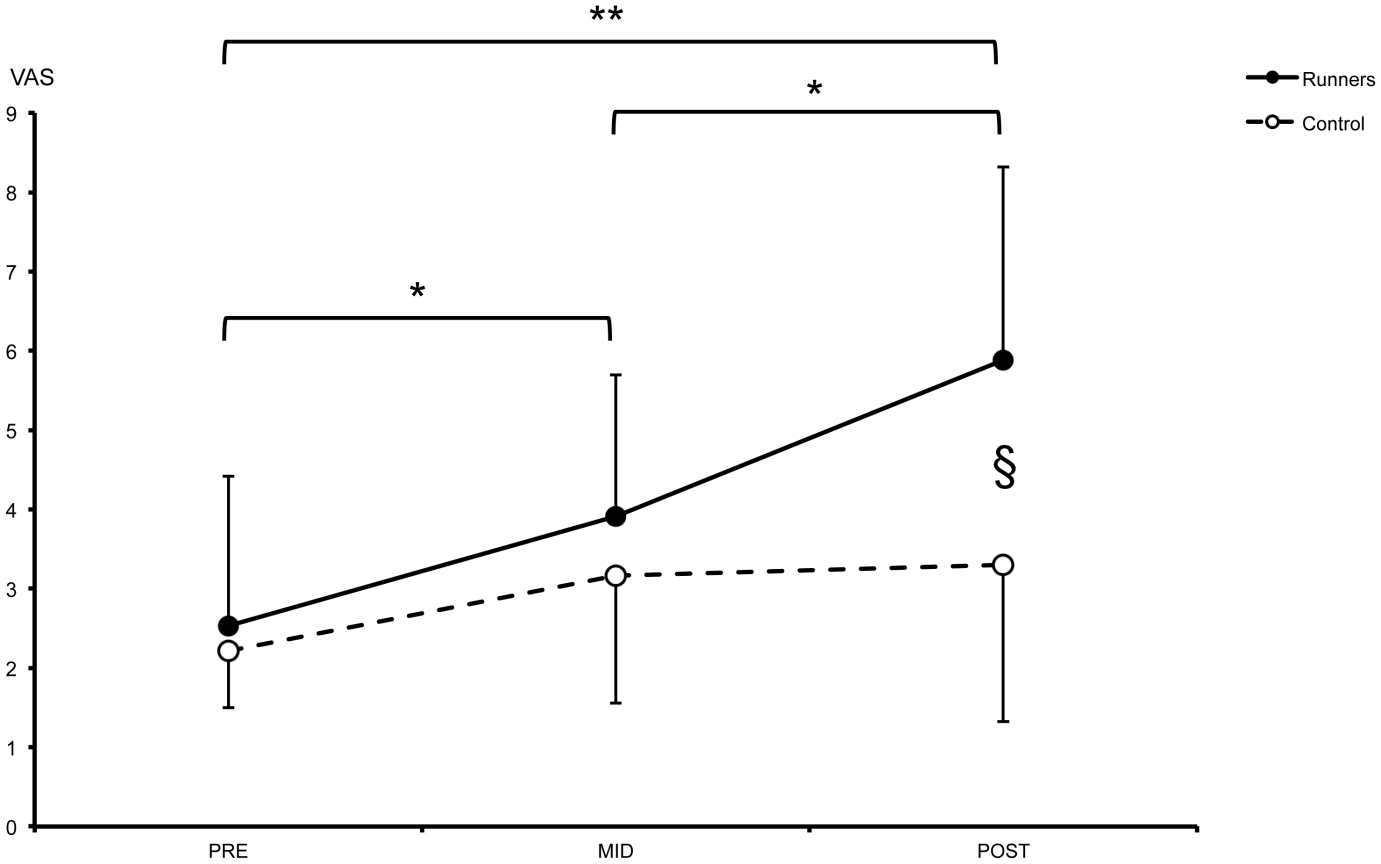
Evolution of the visual analogue scale score in two groups of subjects during the three sessions of measurements during the race.

A significant increase in the perceived level of fatigue was observed for runners between PRE and MID (54±5%; p<0.05), between MID and POST (50±37%; p<0.05) and between PRE and POST (132±29%; p<0.01). A significant difference between runners and controls was only detected at POST (78±23%; p<0.05) (F = 3.454, p = 0.009). These comparisons are presented in Fig. 3.

### Summary statistics of postural control parameters

The summary statistics of these parameters are shown in Table 2.

**Table 2:** Standard postural parameters in EO and EC.

			Eyes Open				Eyes Open		
		*PRE*	*MID*	*POST*		*PRE*	*MID*	*POST*	
Total Length	R	487.43±122.46	590.63±126.24	649.14±163.33	*	687.11±205.85	727.93±211.23	900.11±367.46	* **$**
(mm)	C	530.28±140.9	500.61±95.39	488.44±124.38	#	699.66±138.63	650.56±109.65	618.31±127.78	#
X length	R	258.89±80.87	296.05±67.38	331.94±89.52	*	339.55±121.52	346.67±84.19	433.64±141.17	* **$**
(mm)	C	296.78±96.78	254.79±61.18	250.81±100.65	#	345.79±93.89	312.41±73.64	316.94±104.2	
Y length	R	357.37±81.61	448.01±102.82	484.09±129.37	*	523.97±157.5	564.13±186.33	693.51±328.83	*
(mm)	C	373.1±96.08	375.11±79.02	362.25±74.3	##	533.66±113.42	504.18±95.18	462.87±91.05	#

R  =  Runners; C  =  Control Group;

*p<0.05 compared with PRE;

p<0.05 compared with MID;

^#^ p<0.05,

^##^ p<0.01, compared with RUNNERS.

For runners, a significant increase in total, AP and ML length of *CoP* was observed between PRE and POST sessions for both the EO and the EC conditions (p<0.05), and a significant change was also observed between MID and POST for total and X length of *CoP* in the EC context (p<0.05).

In the EO context, a significant difference was found between runners and controls at POST for all parameters with interaction between group and time for total length of *CoP* (F = 6.159, p = 0.004), AP length of *CoP* (F = 5.009, p = 0.011) and for ML length of *CoP* (F = 5.190, p = 0.009). In the EC context, a significant difference was found between runners and controls at POST for total and Y length of CoP with group-fatigue interaction (F = 6.454, p = 0.001).

In the control group, no significant changes in postural control parameters were observed between experimental sessions and between EO and EC conditions.

### Stabilogram-diffusion analysis

**Figure 4:**
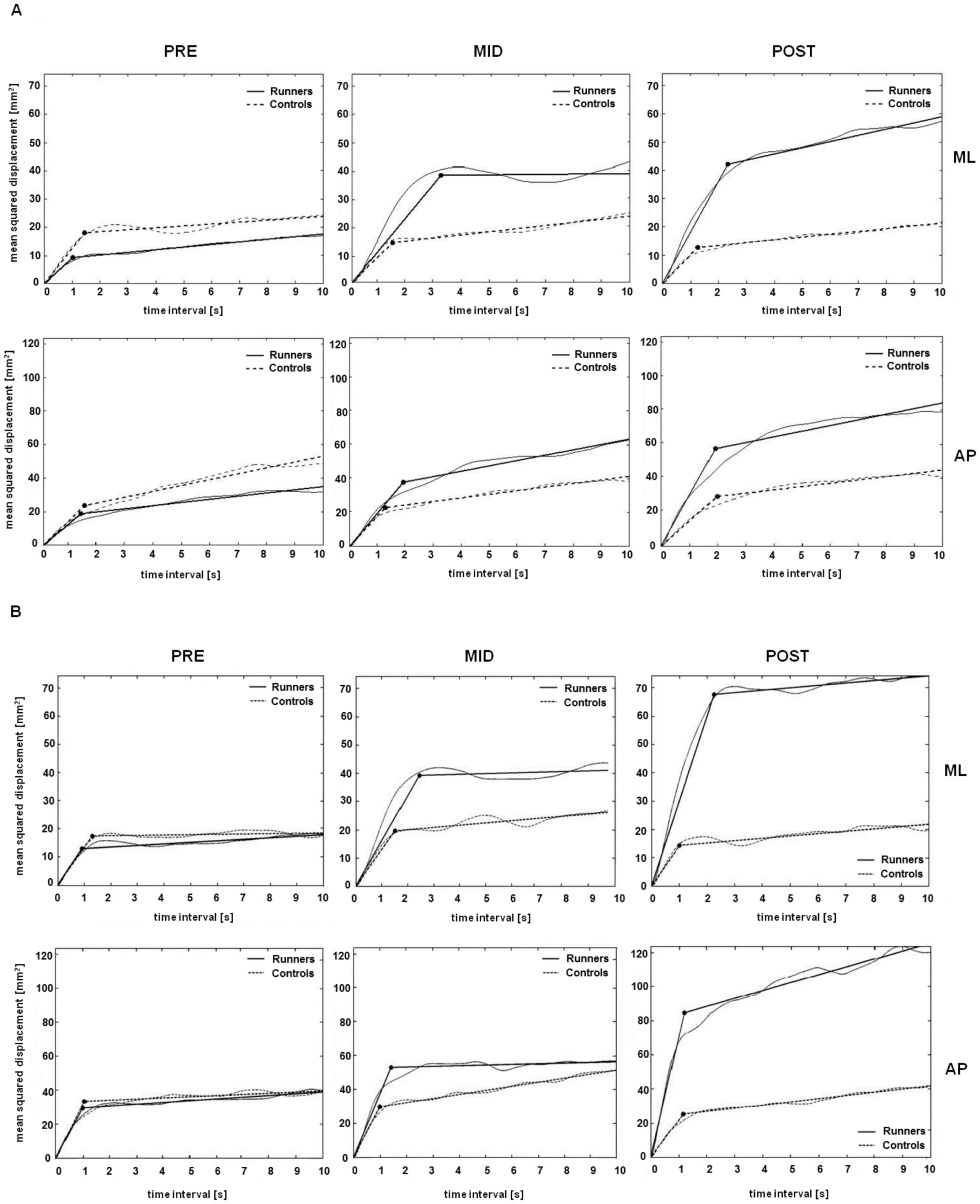
Graphical evolution of Stabilogram-diffusion in the medio-lateral (ML) and antero-posterior (AP) plane for the two groups of subjects during the three sessions of measurements during the race. A: EO condition, B: EC condition.

The same pattern of changes in the SDA parameters was observed for both EO and EC conditions in each group (Fig. 4).

**Figure 5:**
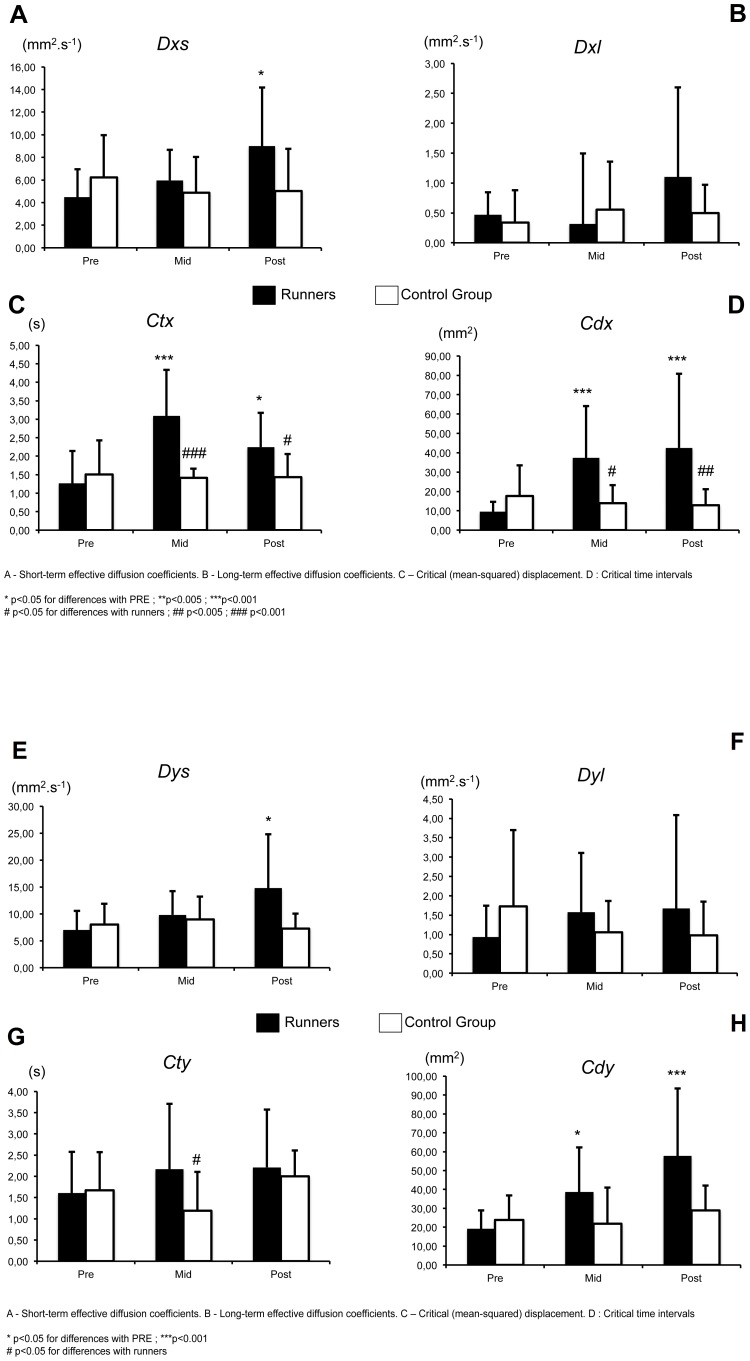
Evolution of Stabilogram-diffusion parameters (EO). A–D. Medio-lateral direction, **E–H.** Antero-posterior direction.

**Figure 6:**
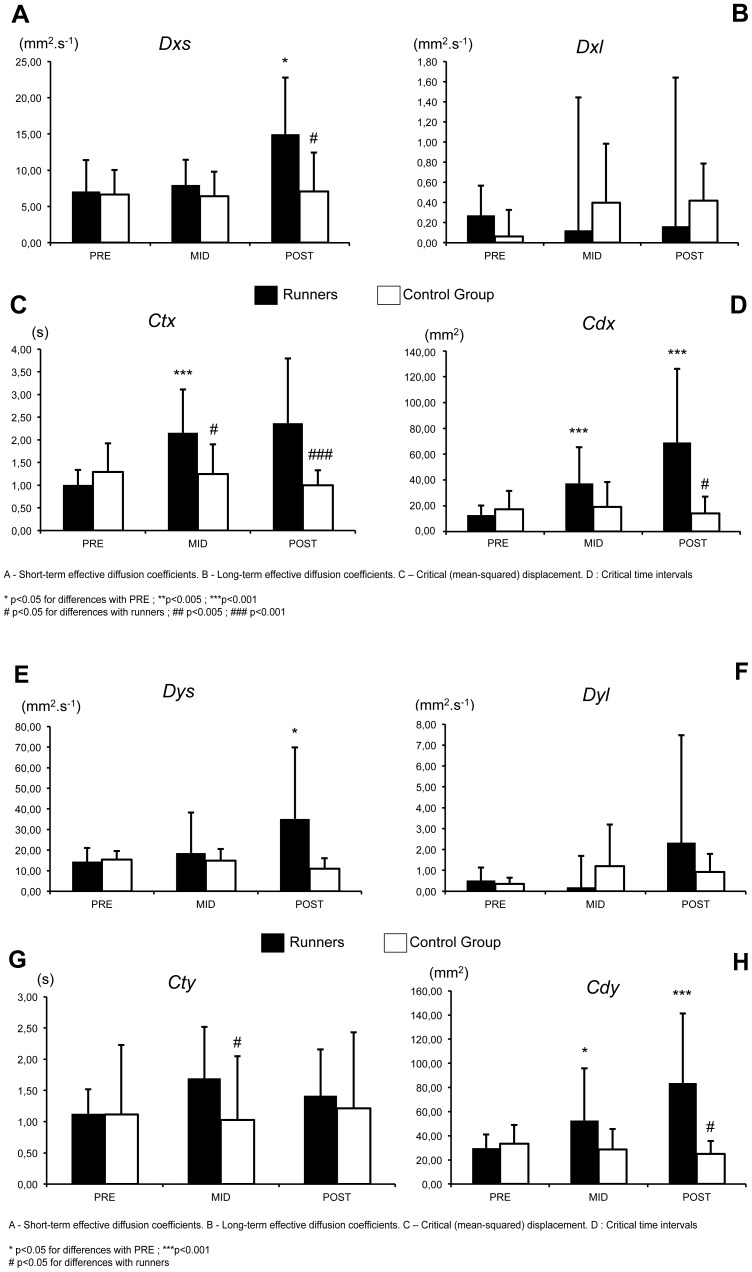
Evolution of Stabilogram-diffusion parameters (EC). A–D. Medio-lateral direction, **E–H.** Antero-posterior direction.

A significantly greater short-term effective stabilogram diffusion was found at POST compared with PRE in the ML (*Dxs*) and AP directions (*Dys*) in runners (Fig 5A, 5E, 6A, 6E). In contrast, the long-term effective diffusion did change between experimental sessions in either runners or controls (Fig 5B, 5F, 6B, 6F).

The critical time interval (*Ctx*) in the ML direction was significantly higher at MID (p<0.001) and POST than at PRE in runners (Fig 5C & 6C). At MID and POST, there was a significant difference between the two groups (F = 12.621, p<0.001).

The critical time interval (*Cty*) in the AP direction did not change in runners or in controls but was different between the groups at MID (Fig 5G & 6G).

The critical (mean-squared) displacement (*Cd*x) in the ML (Fig. 5D & 6D) and in the AP direction (Fig 5H & 6H) was significantly higher at MID and at POST than at PRE for runners. A significant difference in *Cdx* was observed between the groups in the EO condition at MID and POST in the ML direction (Fig 5D) (F = 14.057, p<0.001) and in the EC condition at POST in the ML and AP directions (Fig 6D & 6H) (F = 8.504, p<0.001).

## Discussion

### Effect of sleep deprivation

The effects of SDep on postural sway are due to the reduced levels of alertness [24] and peak when the body temperature reaches its minimum [25]. Neuropsychological data show that after a night of SDep, neuronal activity decreases primarily in the cortico-thalamic network, which mediates attention and higher-order cognitive performance [41]. After a night of SDep, the bilateral posterior-parietal prefrontal areas (PFC) are less activated [41], [42], thereby prompting lower levels of activity in the central executive system. This change can affect executive functions, such as mental flexibility, behavioural inhibition, thinking and problem solving, and is likely to also affect sensorimotor integration [41]. Alterations in postural control due to SDep and muscle fatigue/damage were observed in runners from mid-race to post-race during this extreme MUM. In contrast, the control subjects, who were sleep deprived to the same level, did not show any major alteration in postural control. These data suggest that the postural instability observed in runners may originate predominantly from the MUM-induced peripheral afferents and not from sleep deprivation itself.

Several studies have shown that complete sleep deprivation alters postural control in healthy subjects [23], [27], [43]–[45]. Ma et al. [44] demonstrated that 24 h of SDep could cause feelings of fatigue and affect postural stability, thereby increasing *CoP* length on the stabilogram. This duration of SDep slowed the cortico-thalamic network and resulted in functional changes in attention and in the pre-frontal cortex [41], [42]. A recent study [46] confirmed these observations, indicating that the circadian phase could affect neuromuscular function and, in particular, muscle strength in healthy subjects. This study suggested that neuromuscular function appeared to be more robust than neurobehavioral function when the duration of wakefulness was within normal ranges (i.e., 18.7 h; range of 12–21 h). Throughout the race, fatigue also accumulated with the number of sleepless nights. The period of SDep increased throughout the race. The control group, which did not show a significant alteration in postural control, showed no significant difference in terms of duration of sleep compared with the group of runners at MID and POST. These data suggest that the total amount of SDep was not sufficient to induce changes in postural control. This insufficiency may have resulted from the fact that both runners and control subjects had slept periodically (in periods of 30 min–1 h) instead of being completely, continuously deprived, which could explain the differences in postural control responses compared with previously cited studies. However, even if the SDep does not appear to significantly alter the postural control, it still has an effect on the reduction of attention. This decrease in attention often induces falls. So better management of sleep during a MUM could reduce the risk of falls and injuries for runners.

### Effect of general fatigue

In this study, runners reported a significant increase in general fatigue (VAS) from only mid-race that, as expected, increased further during the second part of the MUM. A local muscular fatigue was observed in runners primarily from MID to the end of the race [47]. Maximal voluntary contraction force decreased slightly at MID (−13±17% and −10±16% for knee extensor and plantar flexor muscles, respectively) and decreased further at POST (−24±13% and −26±19%) in runners, whereas these parameters did not change in the control group [47]. One may hypothesise that the magnitude of general and muscular fatigue, which was not large at MID, was the primary factor underlying the observed alterations in postural control. It is known that any muscular exercise that mobilises or solicits a large part of the body musculature, such as running, induces physiological alterations and important mechanical impacts upon the musculoskeletal system, which are likely to impair the effectiveness of the postural regulating mechanisms [30]. Moreover, the conditions under which the running is performed influence postural control differently. In a review, Paillard et al. [48] demonstrated that a fatigue induced by general exercise could alter sensory integration (visual, proprioceptive and vestibular information). When exercise intensity is greater than the lactate accumulation threshold, the deterioration in postural control becomes significant, whereas at intensities below this threshold, postural control remains undisturbed. However, even at low intensities, one may hypothesise that prolonged, continuous exercise may disturb postural control, which has been confirmed after a long-distance triathlon or prolonged ergocycle exercise (60 min) [49]–[51].

### Effects on postural control – Standard parameters

Many studies have attempted to identify the most relevant variables that can be extracted from the *CoP* trajectory to reveal the underlying control process [5], [52], [53]. In general, posturographic platform data have been analysed to extract traditional postural sway parameters: mediolateral CoP range (mm; ML-sway range); anteroposterior CoP range (mm; AP-sway range); mean velocity of *CoP* sway (mm/s); and sway area (mm^2^) – the elliptical area of the *CoP* points. The standard posturographic parameters were altered significantly in runners at the end of the race only. The total displacement (total length), ML and AP lengths of *CoP* were higher at POST only in runners and were significantly different compared with the control group. At the end of the race, postural parameters were primarily affected in the AP direction (36±14%), with reduced effects in the ML direction (29±18%). Winter et al. [54] proposed that ankle plantar- and dorsi-flexors play a dominant role in the control of AP movements, whereas hip adductors and abductors primarily control ML sway. In our study, it appears that the specificities of this mountain race (large uphill/downhill sections; unstable ground) led to greater muscle fatigue of the ankle plantar than the dorsi-flexor muscles and the hip adductors and abductors. These data are consistent with the observations made by Fourchet et al., who reported greater ankle plantar than dorsi-flexor strength loss after a 5-h hilly run [55].

### Effects on postural control - Stabilogram diffusion analysis

Stabilogram-diffusion analysis (SDA), as described by Collins and De Luca [5], [56], has been performed on the *CoP* trajectories. This analysis focused on the short-term (*Dxs*, *Dys*) and long-term (*Dxl*, *Dyl*) diffusion coefficients and the critical time (*Ctx*, *Cty*) and critical displacement (*Cdx*, *Cdy*), which reflect effective stochastic activity of open-loop and closed-loop postural control mechanisms in the ML and AP directions. These parameters are derived from the slopes of the short-term and long-term regions of the linear stabilogram-diffusion plot. The authors show that *CoP* series correlated positively over the short term (i.e., over short observation times) but correlated negatively over the long term.

In a time-series framework, a positive correlation signifies that an increasing trend in the past is likely to be followed by an increasing trend in the future. In this case, the series is said to be persistent. Conversely, a negative correlation signifies that an increasing trend in the past is likely to be followed by a decreasing trend, and this type of series is said to be anti-persistent. The transition from persistent to anti-persistent correlation patterns over different time scales is known as a “cross-over phenomenon” [57]. Cross-over is typically caused by the variable under study being constrained within given limits [57]. These bounding effects are essential because they suggest that some control, either direct or indirect, is being exerted on the variable under study. Following this line of thought, Collins and De Luca [5], [58] supported the idea that postural control may be position-based. The authors argued that postural sway displacements “*are left unchecked by the postural control system until they exceed some systematic threshold*” [5] and that “*the presence of longer-range negative correlations in the CoP data suggests that closed-loop mechanisms are utilized over long-term intervals of time and large displacements*” [58]. The short-term (*Ds*) and long-term (*Dl*) diffusion coefficients, which characterise the effective stochastic activity of open-loop and closed-loop postural control mechanisms, respectively, are derived from the slopes of the short-term and long-term regions of this plot. It has been suggested that in the short-term region, the postural control system operates in an open-loop mode and does not directly rely on sensory information over short-time intervals, whereas the long-term region reflects closed-loop control mechanisms that the human postural control system operates with sensory feedback [5], [56]. The transition between open-loop and closed-loop control has been termed the critical point, the coordinates of which reflect the average time interval (critical time, *Ct*) and sway displacement (critical displacement, *Cd*) where closed-loop control begins to dominate postural sway. Interpretation of the SDA may offer more insight into the nature of the process controlling the *CoP* trajectories and may be more sensitive than traditional *CoP* measures. Previous research groups have shown that SDA parameters are sensitive to the effects of fatigue [16], [68]. However, one limitation of SDA is the underestimation of long-term correlations (second slope of SDA) and the fact that the assessment, at the individual level, is based on poorly defined diffusion plots due to an evident contamination by low-frequency oscillatory components [59].

Unlike standard *CoP* parameters that were significantly increased only at POST, SDA was already modified at MID. First, at MID, there was a significant increase in *Ctx*, *Cdx*, and *Cdy* in the EO and EC conditions. The general fatigue in runners appears to be one reason underlying these modifications in postural control. These alterations in postural control were significant for SDA, which suggests that the pacing strategies (i.e., a slow pace from the beginning of the race) and SDep, which resulted in very low concentric/eccentric contraction intensity, preserve neuromuscular function despite the apparent extreme difficulty of this event [47]. It also appears that in general, SDA is more sensitive than standard *CoP* parameters. The latter measurements do not consider the dynamic characteristics of the *CoP* motion over the base of support; thus, they are less useful in comprehending the mechanisms used by the central nervous system to control posture. In contrast, SDA parameters can correlate with the steady-state dynamic behaviour of *CoP* motion and quantify short- and long-term correlations that may be explained in terms of motor control theory [5].

Second, the most important alterations in SDA appeared at POST. The fatigue at POST caused an increase in short-term stochastic activity in AP (*Dys*) and ML (*Dxs*), as well as an increased critical displacement in the AP and ML direction (*Cdy* and *Cdx*) and in critical time in the ML direction (*Ctx*). These alterations in postural control appeared to be directly induced by the large increase in neuromuscular fatigue at POST, as observed in the study by Saugy et al. [47]. The greater values of *Dys* and *Cdy* at POST indicate an increase in stochastic activity during short-term intervals, when sway trends to drift from an equilibrium point in an open-loop control mode, and a higher sway displacement prior to when closed-loop feedback mechanisms dominate sway behaviour, respectively. Because the subjects performed the postural tasks, it is likely that flexor proprioception (muscle spindles and golgi tendon organs) played an important role in the regulation of postural control. Consequently, a decrease in performance could be due to fatigue affecting proprioceptive feedback [60], which would be consistent with an increase in critical displacement (*Cdy*) in the AP and ML directions because it would indicate a decrease in sensitivity of the postural control loop and a lower quality control of across-time intervals when closed-loop control dominates behaviour. These changes in the temporal interaction of open- and closed-loop control mechanisms are supported by observations of increased reflex time [61], reduced proprioception [62] and weaker muscle strength [63] that have been reported as common consequences of aging and the fatigue process.

It is known that both extrinsic muscles, (e.g., tibialis posterior, tibialis anterior, peroneous longus and peroneous tertius) and plantar intrinsic foot muscles (e.g., abductor hallucis, flexor hallucis brevis, flexor digitorum brevis, abductor digiti minimi and dorsal interossei) provide dynamic support of the foot medial longitudinal arch [64], [65] not only during gait but also while in static stance [66]. During MUM, great fatigue of the plantar flexors has been reported, whereas in dorsi-flexors [31], [47], [55], the magnitude of fatigue appears less [55]. This increased fatigue-induced strength imbalance likely influences the control of AP movements. However, we did not observe any correlation between the Pre-to-Post changes in the PF neuromuscular characteristics [47] and the posturographic values.

There are additional physiological mechanisms that could explain these findings. Reports have shown impaired muscle-spindle sensitivity following prolonged exercise in animals [67], [68] that may be due to the influence of metabolites or/and inflammatory substances [68] or the modulation of reflex pathways originating from small-diameter muscle afferents [69]. Furthermore, an increase in short-term postural sways during quiet standing could be caused by increased agonist-to-antagonist co-activation. Laughton et al. [70] reported that short-term postural sway was correlated significantly with co-activity of ankle muscle. Greater co-activation may assist in enhancing joint proprioception by increasing the firing rate and by recruiting primary afferents, thereby enhancing the functional behaviour of the associated closed-loop postural control mechanism.

An additional factor may cause the peripheral effect of muscle fatigue induced by ultratrail racing. The metabolic milieu of the muscle is altered during exercise-induced fatigue, and the capacity of the system to provide fast and accurate contraction decreases. Thus, the neuromuscular system becomes unable to maintain constant and accurate muscle tension. This peripheral fatigue was reported by Saugy et al. [47], and Gribble et al. [11], [12] claimed that compensation occurs in these fatigued states, which results in overcompensations that, in turn, cause larger displacements of *CoP*.

Our study also revealed a significant increase in *Ctx* (critical time in ML plan) in the EO and EC conditions; thus, runners take longer at POST than at MID to stabilise their bodies using sensory inputs from the environment. This alteration was not found in the anteroposterior plane and could have been caused by the terrain. Indeed, the trails used by the runners in the mountains are often narrow, which requires constant adaptation of their dynamic balance and may tire the adductor and abductor muscles of the hip. Indeed, Gribble et al. suggested that when these muscles are tired, postural control in the ML plane deteriorates [11], [12].

Some limitations of our study must be considered. The first limitation is the age difference between the control and runner groups. It is known that postural control declines with increasing age. Thus, in theory, the age difference of 15 years (29 vs. 44 yrs.) may limit our findings. However, we believe that this difference did not influence the results because both groups were healthy adults without any declared pathology, and the age difference was relatively small. Postural alterations are marked in patients with associated pathologies and in the elderly [71]. Another potential limitation of our study is the 30-minute delay between the arrival of the runners (to the finish line) and the POST tests. However, compared with the mean duration of the MUM of 127 h, this delay appears negligible.

To preserve the kinesthetic and proprioceptive capital during the race, it seems important that the riders can regularly change shoes and limited joint shocks that could damage certain receptors that are responsible for optimal postural control.

In conclusion, this study showed significant effects of fatigue, induced by the world's most challenging MUM, on alterations in postural control in runners. Because controls with the same level of SDep showed minimal alterations in balance, this change originates primarily from muscle fatigue. It is likely that the mountainous characteristics of the MUM (unstable ground, primarily uphill/downhill running, and altitude) increase this fatigue and therefore the difficulty in maintaining balance. Overall, these results indicate very clearly that at the end of this extreme MUM, it appears very difficult for the body to control motor tasks using all sensorimotor afferents. One may speculate that postural control could be used as an index of fatigue. The stabilogram diffusion analysis appears to be more accurate and sensitive that standard parameters but therefore complementary. SDA analysis indicated effects of fatigue across short-time intervals, where open-loop control dominates behaviour, and over long-term intervals, where closed-loop control mechanisms dominate.

Another practical benefit of assessing postural control in MUM races is the potential correlation between postural control alterations and injuries. Moreover, further study is required to assess postural control parameters, which appear to be of practical/safety interest, in long distance races on narrow mountain paths.
