# Correction: Alterations in Postural Control during the World's Most Challenging Mountain Ultra-Marathon

**DOI:** 10.1371/journal.pone.0093528

**Published:** 2014-03-21

**Authors:** The PLOS ONE Staff

The sixth author’s name is spelled incorrectly. The correct name is: Grégoire Millet.

There is an error in Table 2. The right-hand column should read “Eyes Closed.” Please see the corrected Table 2 below:

**Table 2:** Standard postural parameters in EO and EC.

		Eyes Open				Eyes Closed			
		*PRE*	*MID*	*POST*		*PRE*	*MID*	*POST*	
									
Total Length (mm)	R	487.43 ± 122.46	590.63 ± 126.24	649.14 ± 163.33	*	687.11 ± 205.85	727.93 ± 211.23	900.11 ± 367.46	*$
	C	530.28 ± 140.9	500.61 ± 95.39	488.44 ± 124.38	**#**	699.66 ± 138.63	650.56 ± 109.65	618.31 ± 127.78	#
									
X length (mm)	R	258.89 ± 80.87	296.05 ± 67.38	331.94 ± 89.52	*	339.55 ± 121.52	346.67 ± 84.19	433.64 ± 141.17	*$
	C	296.78 ± 96.78	254.79 ± 61.18	250.81 ± 100.65	**#**	345.79 ± 93.89	312.41 ± 73.64	316.94 ± 104.2	
									
Y length (mm)	R	357.37 ± 81.61	448.01 ± 102.82	484.09 ± 129.37	*	523.97 ± 157.5	564.13 ± 186.33	693.51 ± 328.83	*
	C	373.1 ± 96.08	375.11 ± 79.02	362.25 ± 74.3	**##**	533.66 ± 113.42	504.18 ± 95.18	462.87 ± 91.05	#

R = Runners; C = Control Group

* p<0.05 compared with PRE

$ p<0.05 compared with MID

# p<0.05, ##p<0.01, compared with RUNNERS

There are errors in Figure 5 and Figure 6. In both figures, there are repeated descriptions for the histograms A, B, C and D and the descriptions for E, F, G and H are missing.

Please see the corrected Figure 5 below:

**Figure 5:**
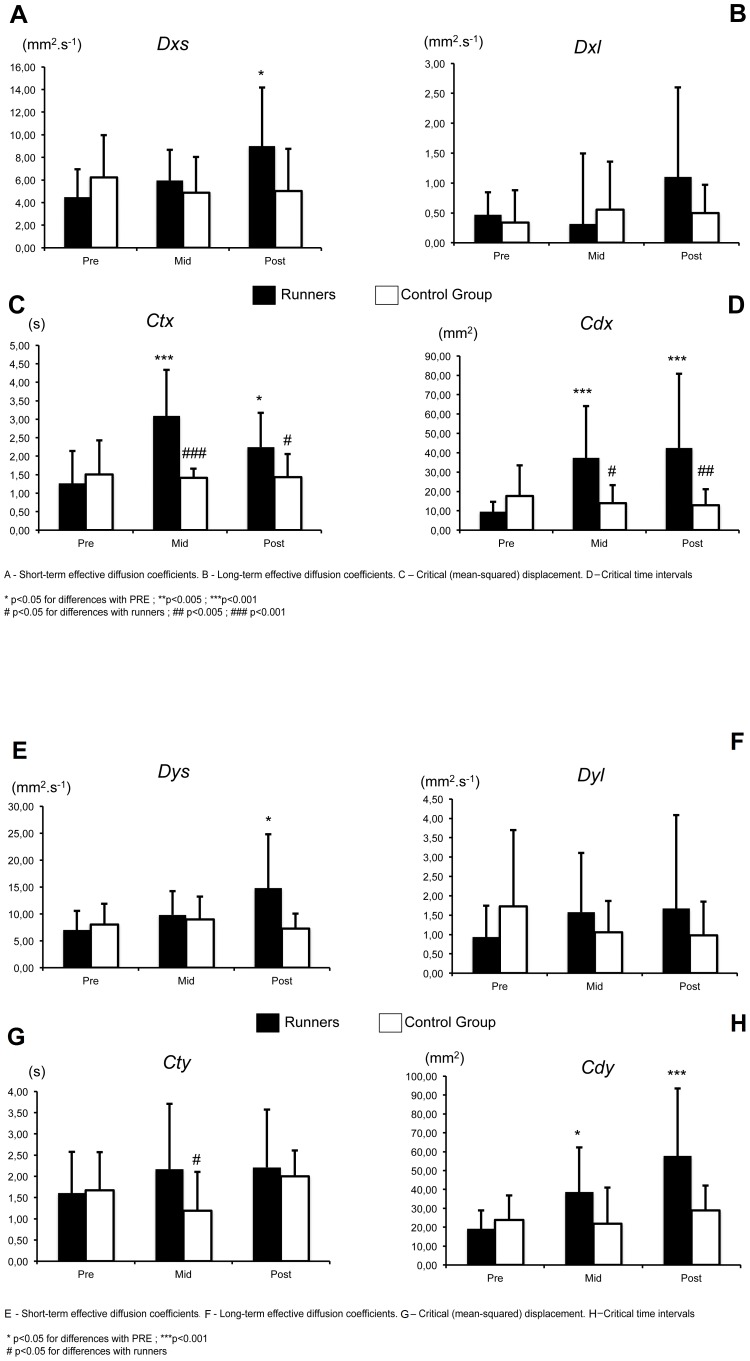
Evolution of Stabilogram-diffusion parameters (EO). A–D. Medio-lateral direction, **E–H.** Antero-posterior direction.

Please see the corrected Figure 6 below:

**Figure 6:**
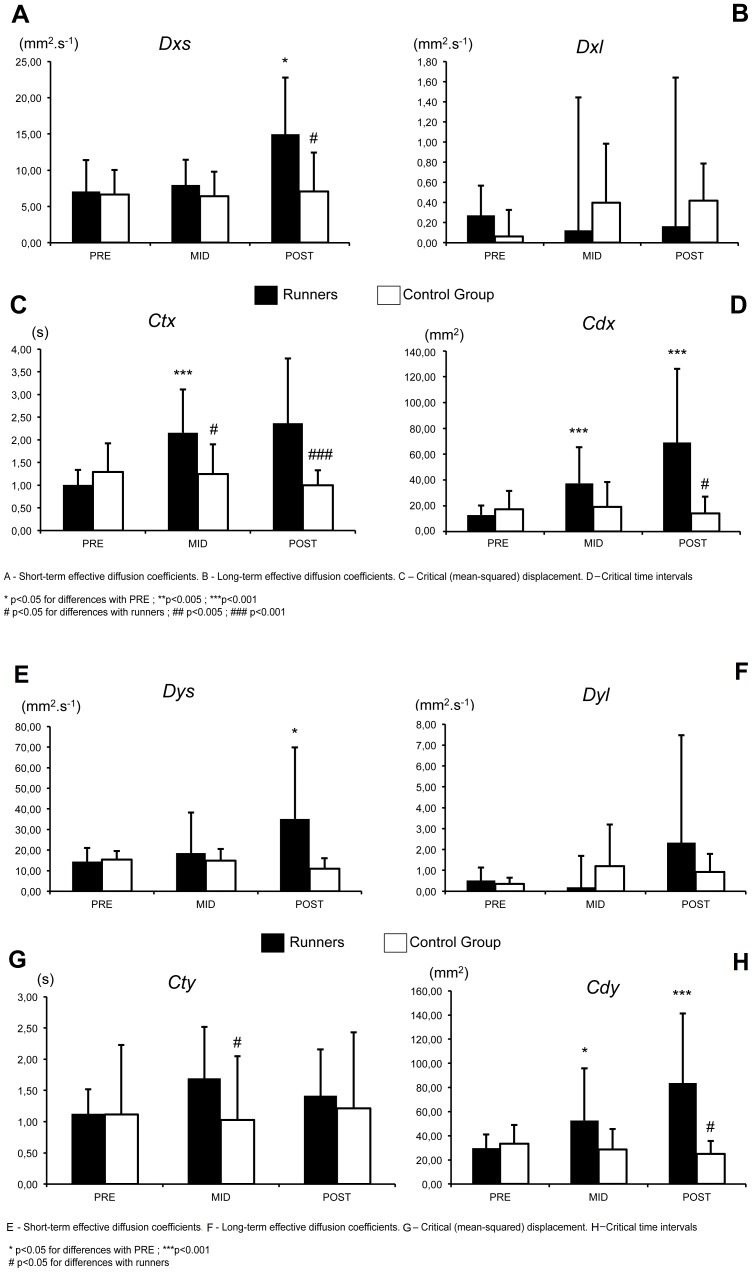
Evolution of Stabilogram-diffusion parameters (EC). A–D. Medio-lateral direction, **E–H.** Antero-posterior direction.

Reference 30 is incorrect. The correct reference should read:

Saugy J, Place N, Millet GY, Degache F, Schena F, et al. (2013) Alterations of neuromuscular function after the World most challenging mountain ultra-marathon. PLoS ONE 8(6): e65596. doi: 10.1371/journal.pone.0065596.
